# Supplementary material for: Biodiversity of Secondary Metabolites Compounds Isolated from Phylum Actinobacteria and Its Therapeutic Applications
Source: Molecules. 2021 Jul 26;26(15):4504. doi: 10.3390/molecules26154504 (PMC8347454; doi:10.3390/molecules26154504)
Supplement: Supplementary file 1 [file molecules-26-04504-s001.zip › molecules-1295167-supplementary.pdf]

Supplementary Material

Review

Biodiversity of Secondary Metabolites Compounds Isolated from Phylum Actinobacteria and Its Therapeutic Applications

Muhanna Mohammed Al-shaibani [1,2], Radin Maya Saphira Radin Mohamed [1,*], Nik Marzuki Sidik [3,*], Hesham Ali El Enshasy [4,5], Adel Al-Gheethi [1,*], Efaq Noman [6], Nabil Ali Al-Mekhlafi [7,8] and Noraziah Mohamad Zin [2]

1 Micro-Pollutant Research Centre (MPRC), Faculty of Civil Engineering and Built Environment, Universiti Tun Hussein Onn Malaysia, Parit Raja 86400, Johor, Malaysia; muhanna@uthm.edu.my
2 Center for Diagnostic, Therapeutic and Investigative Studies, Faculty of Health Sciences, Universiti Kebangsaan Malaysia, Kuala Lumpur 50300, Malaysia; noraziah.zin@ukm.edu.my
3 Faculty of Agro-Based Industry, Universiti Malaysia Kelantan, Jeli 17600, Kelantan, Malaysia
4 Institute of Bioproducts Development (IBD), Universiti Teknologi Malaysia (UTM), Skudai 81310, Johor, Malaysia; henshasy@ibd.utm.my
5 City of Scientific Research and Technology Applications (SRTA), 21934 New Burg Al Arab, Alexandria, Egypt
6 Applied Microbiology Department, Faculty of Applied Sciences, Taiz University; Taiz 6803, Yemen; hw160052@siswa.uthm.edu.my
7 Atta-ur-Rahman Institute for Natural Product Discovery, UiTM, Puncak Alam Campus, Bandar Puncak Alam 42300, Selangor, Malaysia; nabilali7@tu.edu.ye
8 Biochemical Technology Program, Department of Chemistry Faculty of Applied Science, Thamar University, Thamar P.O. Box 87246, Yemen
* Correspondence: maya@uthm.edu.my (R.M.S.R.M.); nikmarzuki@umk.edu.my (N.M.S.); adel@uthm.edu.my (A.A.-G.)

Abbreviations: N.C: Not classified; MDRB, Multidrug resistant bacteria. S. *Streptomyces*, (PK) polyketide

Table S1. Historically isolation of bioactive compounds from Actinobacteria

Compound	Producer	Source	Uses	Reference
Actinomycin D (1)	*Streptomyces*	Soil bacteria	Anticancer	[1]
Streptomycin (2)	*Streptomyces*	Soil bacteria	Anti T.B	[2]
Gramicidin (3)	*Streptomyces*	Soil bacteria	Antimicrobial	[3]
Chloramphenicol (CAP) (4)	*S. venezuelae*	Soil bacteria	Antimicrobial	[4]
	S.omiyaensis	Soil bacteria	Antimicrobial	[5]
	Streptosporangium viridogriseum var kofuense	Soil bacteria	Antimicrobial	[6]
Cephamycins C (5)	actinomycetes isolated from soil	Soil bacteria	Antimicrobial	[7]
	S. lactamdurang	Soil bacteria	Antimicrobial	[8]
Fumaramidmycin (6)	*S. kurssanovii*	soil bacteria	Antimicrobial	[9]
Crisamicins C (7)	*Micromonospora purpurea chromogenes*	soil bacteria	Antimicrobial	[10].
Polyene Antibiotics (PA-5 And PA- 7 (8)	*Streptoverticillium sp. 43/16*	soil bacteria	Antimicrobial	[11]
Phospholine (10)	*S.hygroscopicus.*	soil bacteria	Anticancer	[12]
Simocyclinones D4 (11 A) Simocyclinones D8 (11 B)	*S.antibioticus Tii 6040*	soil bacteria	Anticancer& Antimicrobial	[13]
Fistupyrone (12)	*streptomyces sp.TP*	soil bacteria	Anti. Alteraniria baeassicicila	[14]
Streptocidins A-D (13)	*Streptomyces sp. Tü 6071*	soil bacteria	Antimicrobial	[15]
Cedarmycin A (14-A) B (14-B)	*S. TP- A0456*	twig of cedar	Antimicrobial	[16]
Kakadumycin A (15)	*S.sp. NRRL 30566*	soil bacteria	Anti.MRSA	[17]
Munumbicins E-4 And E-5 (16)	*S. NRRL3052*	soil bacteria	Anti.MRSA	[18]

Abbreviations: N.C: Not classified; MDRB, Multidrug resistant bacteria. S. *Streptomyces*, (PK) polyketide

Table S2. List of antifungal, growth promoting, antitumor and antiparasitic bioactive compounds, chemical classification and their application which isolated from Actinobacteria.

Antifungal	Producer	Chemical class	References
Candicidin	*S. griseus*	polyene macrolide	[19]
Natamycin	*S. nataensis*	tetraene polyene	[20]
Nystatine	*S. noursei*	polyene macrolide	[21]
Polyoxins	*S. cacaoi var asoensis*	Nucleoside peptide	[22]
Avermectin	*S. avermitilis*	Macrolide	[23]
Hygromycin B	*S.hygroscopicus*	Aminoglycoside	[24]
Milbemycin	*S. argilaceus*	Macrolide	[25]
Amphotericin B	*S. nodosus.*	PKS*	[26]
Lasalocid	*S. lasaliensis*	Polyether	[27]
Monensin	*S. cinnamonesis*	Polyether	[28]
Bambermycin	*S. bambergiensis*	Aminoglycoside	[29]
Nosiheptide	*S. actuosus*	Thiopeptide	[30]
Sterptothricin	*S. lavendulae*	N-Glycoside	[31]
Thiostrepton	*S. azureus*	Thiopeptide	[32]
Tylosin	*S. fradiae*	Macrolide (PK)*	[33]
Virginamycin	*S. virginiae*	(PK*)	[34]
Salinomycin	*S. albus*	Polyether (PK*)	[35]
Actinomycin D	*Streptomyces lannensis* T1317-0309	Peptide	[36]
Adriamycin	*S. peucetius*	Anthracycline	[37]
Bleomycin	*S. verticillus*	Glycopeptide	[38]
Mithramycin	*S. argilaceus*	Aureolic acid	[39]
Mitomycin C	*S. caespitosus*	Benzoquinone	[40]
Actinomycins D	S. sp. ZZ338	polypeptide	[41]

Abbreviations: N.C: Not classified; MDRB, Multidrug resistant bacteria. S. *Streptomyces*, (PK) polyketide

Table S3. Terrestrial and rhizosphere Actinobacteria isolation and screening for their antimicrobial activity.

Bioactive Compound	Producer	Chemical Class	Bioactivity	Source of isolation	References
Cyclohexane, Butyl Propyl Ester, And 2,3-Heptanedione.	*S.* SUK 08	N. C	Antimalarial Drug	Soil	[42]
cyclo-(tryptophanyl-prolyl) chloramphenicol.	*S.* SUK 25	Diketopiperazine Protein Inhibitor	Anti- MRA	Soil. *Zingiber spectabile*	[43]
cyclo-(L-Val-L-Pro), *cyclo*-(L-Leu-L-Pro), *cyclo*-(L-Phe-L-Pro), *cyclo*-(L-Val-L-Phe), and N-(7-hydroxy-6-methyl-octyl)-acetamide	*S.* SUK 25	Diketopiperazine	Anti- MRA	Soil. *Zingiber spectabile*	[44]
Several compounds	S. SCA3-4	N. C	MDRB &Pathogenic filamentous fungi	rhizosphere soil, China	[45]
Unknown	*S. violaceoruber* B263 UFL, *S. albus* B262 UFL *S. badius B192 UFL*	N. C	MDRB &Pathogenic filamentous fungi	Sahara areas, south of Algeria	[46]
Actinomycin D	*Streptosporangium* sp. (AI-21).	N. C	Anti-tumor and antibiotic	Soil, Uttarakhand, India	[47]
Unknown	eighty-six actinomycete	N. C	MDRB	Soil, Aquilla Safari, South Africa.	[48]
Unknown	*S.* DV1S and GR9a-5	N. C	MDRB	Soil, Uttarakhand hills	[49]
Cyclo (S-Pro-S-Val)	AGM12-1	Diketopiperazine	Anti-tumor properties	Soil, Egypt	[50]
Unknown	CA-02, CA-06, CA-07, and CA-17	phenolic and flavonoid	antioxidant activity	Rhizosphere. Brazilian Caatinga	[51]
Unknown	Kocuria kristinae, Kocuria rosea, S. griseus, *S. flaveolus* Actinobacteria	N. C	MDRB &Pathogenic filamentous fungi	soil samples of Egypt	[52]

Abbreviations: N.C: Not classified; MDRB, Multidrug resistant bacteria. S. *Streptomyces*, (PK) polyketide

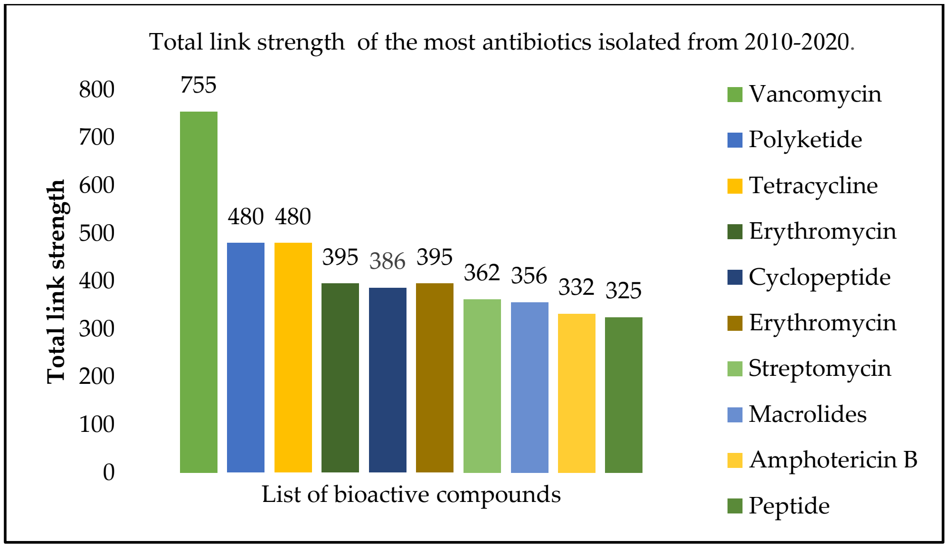

Figure S1. The spread of reviewed and cited papers based on the scattered keywords of the antimicrobial isolated from 2010-2020 from phylum Actinobacteria.

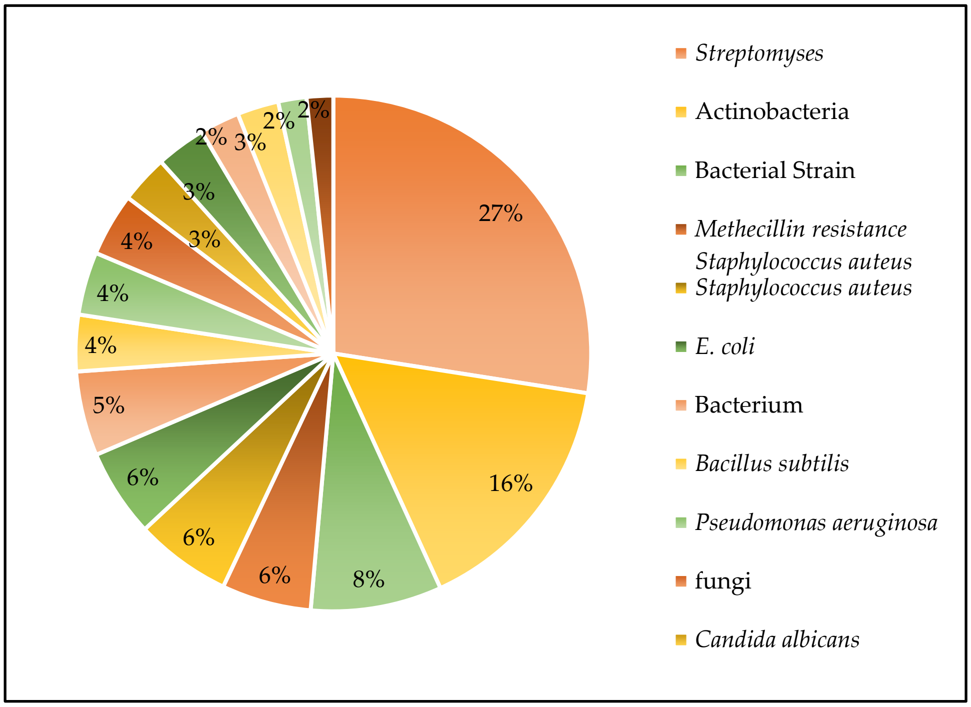

Figure S2. Percentage of diversity of keywords from the Scopus database for Actinobacteria, *Streptomyces*, natural products, primary, secondary metabolites, habitat effects of environments, pharmaceutical industry using VOSviewer software tool to analyse and visualise scientific literature.

Abbreviations: N.C: Not classified; MDRB, Multidrug resistant bacteria. S. *Streptomyces*, (PK) polyketide

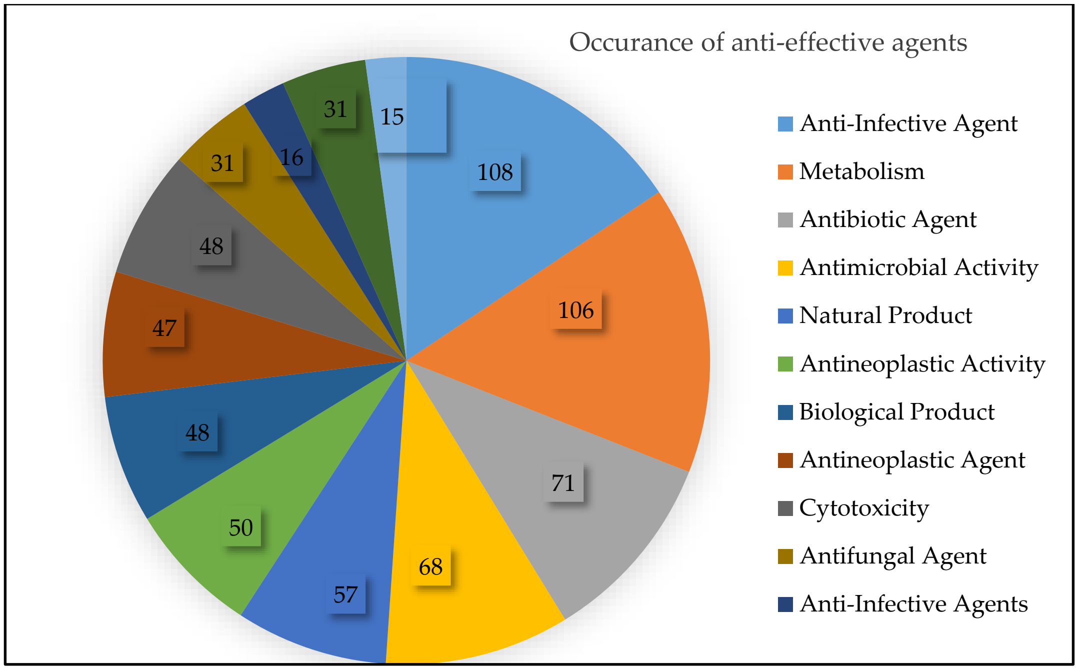

Figure S3. Spread of reviewed and cited papers based on the dispersed keywords of anti-inflective agents' occurrence. Data was extracted using VOSviewer software to analyse and visualise scientific literature.

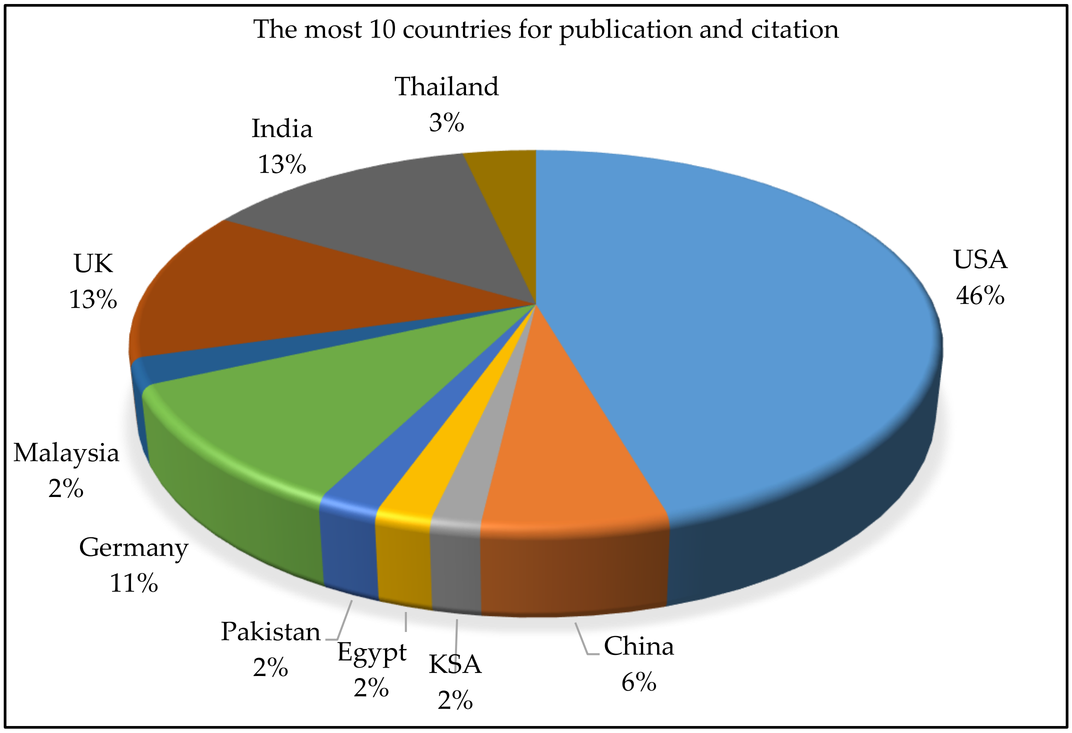

Figure S4. Spread of reviewed and cited papers based on the scattered keywords of the countries for the publication and citation. Data was extracted using VOSviewer software.

Abbreviations: N.C: Not classified; MDRB, Multidrug resistant bacteria. S. *Streptomyces*, (PK) polyketide
